# Activation of Nrf2 by Electrophiles Is Largely Independent of the Selenium Status of HepG2 Cells

**DOI:** 10.3390/antiox10020167

**Published:** 2021-01-23

**Authors:** Sarah Tauber, Maria Katharina Sieckmann, Katrin Erler, Wilhelm Stahl, Lars-Oliver Klotz, Holger Steinbrenner

**Affiliations:** 1 Institute of Nutritional Sciences, Nutrigenomics Section, Friedrich Schiller University Jena, D-07743 Jena, Germany; sarah.tauber@uni-jena.de (S.T.); ka.sieckmann@yahoo.de (M.K.S.); katrin.erler@uni-jena.de (K.E.); lars-oliver.klotz@uni-jena.de (L.-O.K.); 2 Institute of Biochemistry and Molecular Biology I, Medical Faculty, Heinrich Heine University Düsseldorf, D-40001 Düsseldorf, Germany; wilhelm.stahl@hhu.de

**Keywords:** selenoprotein, GPX, TXNRD1, HMOX1, SQSTM1, GSTA1, oxidative stress, cardamonin, sulforaphane, DEM

## Abstract

Selenoenzymes, whose activity depends on adequate selenium (Se) supply, and phase II enzymes, encoded by target genes of nuclear factor erythroid 2-related factor 2 (Nrf2), take part in governing cellular redox homeostasis. Their interplay is still not entirely understood. Here, we exposed HepG2 hepatoma cells cultured under Se-deficient, Se-adequate, or Se-supranutritional conditions to the Nrf2 activators sulforaphane, cardamonin, or diethyl maleate. Nrf2 protein levels and intracellular localization were determined by immunoblotting, and mRNA levels of Nrf2 target genes and selenoproteins were assessed by qRT-PCR. Exposure to electrophiles resulted in rapid induction of Nrf2 and its enrichment in the nucleus, independent of the cellular Se status. All three electrophilic compounds caused an enhanced expression of Nrf2 target genes, although with differences regarding extent and time course of their induction. Whereas Se status did not significantly affect mRNA levels of the Nrf2 target genes, gene expression of selenoproteins with a low position in the cellular “selenoprotein hierarchy”, such as glutathione peroxidase 1 (GPX1) or selenoprotein W (SELENOW), was elevated under Se-supplemented conditions, as compared to cells held in Se-deficient media. In conclusion, no major effect of Se status on Nrf2 signalling was observed in HepG2 cells.

## 1. Introduction

Activation of the transcription factor nuclear factor erythroid 2-related factor 2 (Nrf2) represents a key mechanism in adaptive responses to environmental challenges elicited by reactive oxygen species (ROS), xenobiotics, and fluctuations in nutrient supply [1,2,3,4]. Thus, Nrf2 controls the gene expression of a large number of proteins, primarily enzymes involved in antioxidant protection and the maintenance of redox homeostasis, in the metabolism and detoxification of xenobiotics, as well as in autophagy and in the metabolism of glucose and lipids [3,4]. Nrf2 exerts its activity as a transcription factor through binding, as a heterodimer together with small musculo-aponeurotic fibrosarcoma (sMaf) protein, to antioxidant/electrophile responsive element (ARE/EpRE) sequences in the promoter region of its target genes. Under basal non-stressed conditions, Nrf2 is usually found at low levels in the cytoplasm, where newly synthesized Nrf2 binds to its main repressor Kelch-like ECH-associated protein 1 (Keap1). Keap1-bound Nrf2 is subjected to poly-ubiquitination by the E3 ubiquitin–protein ligase cullin-3 (CUL3) and to rapid proteasomal degradation [1,2,3,4]. Therefore, Keap1 has been proposed to act as a “floodgate” that largely blocks the entry of Nrf2 into the nucleus under non-stressed conditions [4,5]. Secondary to the inhibitory role of Keap1, Nrf2 can also become poly-ubiquitinated and degraded following its phosphorylation by glycogen synthase kinase-3β (GSK-3β); proteasomal degradation of Nrf2 through this pathway is suppressed by agents that activate protein kinase B (PKB)/Akt, which in turn phosphorylates and thus inactivates GSK-3β [3,4]. Primarily, activation of Nrf2 occurs through ROS and electrophiles, inducing oxidation of and adduct formation with specific cysteine residues in Keap1. Under such stressed conditions, the “floodgate” Keap1 opens: Nrf2 no longer binds to Keap1, resulting in its stabilization and nuclear translocation [1,2,3,4]. The activation of Nrf2 is fostered through a positive feedback loop: Nrf2 upregulates the gene expression of sequestosome-1/p62, a protein that is capable of competitively binding to Keap1 and conveying it to autophagic degradation [6,7].

There is some cross-talk between Nrf2-mediated signalling and other molecular pathways that contribute to sensing and detoxification of ROS and/or xenobiotics, such as those involving the aryl hydrocarbon receptor (AhR), the forkhead box class O (FoxO), and the peroxisome proliferator-activated receptor-gamma (PPAR-γ) transcription factors [2,3,8]. To efficiently cope with oxidative stress, the essential trace element and micronutrient selenium (Se) is required as well. In form of the amino acid selenocysteine (Sec), Se is incorporated into 25 human selenoproteins, including several antioxidant selenoenzymes such as glutathione peroxidases (GPx), thioredoxin reductases (TrxR), and methionine sulfoxide reductase B1 (MsrB1) [9,10]. Interestingly, mRNA levels of two selenoenzymes, GPx2 and TrxR1, were previously shown to be elevated upon activation of Nrf2 [11,12]; moreover, synergistic induction of GPx2 and/or TrxR1 by the Nrf2-activating electrophilic compound sulforaphane (SFN) and Se was shown to protect Caco-2 human colorectal adenocarcinoma cells and HepG2 human hepatoma cells against oxidative stress-mediated cell death [13,14]. On the other hand, a compensatory induction of Nrf2 signalling and expression of Nrf2 target genes were observed under experimental conditions of defective selenoprotein biosynthesis, caused either by dietary Se deficiency or by genetic disruption of ribosomal selenoprotein translation [15,16,17,18]. In particular, Nrf2 activation was reported following knock-down or inhibition of the selenoenzyme TrxR1 [19], al-though two recent studies did not corroborate the expected general activation of Nrf2 signalling in response to Se deficiency, as Nrf2 target genes were not upregulated in the liver of mice fed a Se-deficient diet in comparison to mice supplied with adequate or supranutritional Se [20,21]. In addition, two studies using cultured mouse lung epithelial cells and Caco-2 cells did not find a synergistic upregulation of Nrf2 target genes by treatment with the inorganic Se compound sodium selenite and the Nrf2 activators auranofin or cardamonin (CAR), respectively [22,23].

These controversial data on the interplay of Nrf2 and Se prompted us to investigate the influence of Se status on Nrf2 signalling in HepG2 cells, a liver-derived human cell line. HepG2 cells cultured under Se-deficient, Se-adequate, or Se-supranutritional conditions were treated with three electrophilic compounds known to induce Nrf2 target genes: the isothiocyanate SFN [13,24], the flavonoid CAR [22,25], or the maleic acid ester diethyl maleate (DEM) [24,26]. SFN and DEM activate Nrf2 through binding to cysteine-151 of Keap1, interrupting the Keap1–Nrf2 interaction [4,24]. The precise mode of Nrf2 activation by CAR has to be determined yet. As CAR contains an α,β-unsaturated carbonyl structure [22], it is likely to undergo a Michael reaction with cysteine residues in Keap1, as it is known for some other phytochemicals such as curcumin [1].

Here, we found that each of the three applied electrophiles caused rapid stabilization and nuclear accumulation of Nrf2 in HepG2 cells, whereas Se deficiency alone was not sufficient to induce Nrf2. Moreover, the cellular Se status exerted only a minor effect on the upregulation of Nrf2 target genes induced by the electrophiles.

## 2. Materials and Methods 

### 2.1. Chemicals

CAR, DEM, SFN, sodium selenite, Neutral Red, thiazolyl blue tetrazolium bromide (MTT), and Ponceau S were from Sigma-Aldrich (Deisenhofen, Germany). Dimethyl sulf-oxide (DMSO) and the reagents for SDS-PAGE were purchased from Carl Roth (Karlsruhe, Germany).

### 2.2. Culture and Treatment of HepG2 Cells

HepG2 cells, obtained from the German Collection of Microorganisms and Cell Cultures (DSMZ; Braunschweig, Germany), were routinely cultured as previously described [26,27] and used between passage 10 and 32 upon receipt. For the experiments, the cells were seeded in 6-well (1 × 10^6^ cells/well) plates (Sarstedt; Nürnbrecht, Germany) and grown to ~75% confluency. Thereafter, the cells were cultured for 16 h (overnight) in serum-free medium, either without the addition of selenium (Se-deficient conditions) or with supplementation of 0.1 µM selenite (Se-adequate conditions) or 1 µM selenite (Se-supranutritional conditions). Then, the cells were incubated for the indicated time periods in serum-free medium containing Se at one of the above concentrations and CAR (50 µM), DEM (1 mM), or SFN (50 µM). Stock solutions of sodium selenite and of the electrophilic compounds were prepared in bi-distilled water and DMSO, respectively. Serum-free culture medium with 0.1% DMSO served as solvent control. The viability of cells was assessed by MTT or Neutral Red assays, according to standard procedures.

### 2.3. RNA Isolation and Real-Time RT-PCR (qRT-PCR) Analysis

RNA isolation and qRT-PCR were performed as previously described [26]. In brief, total RNA was isolated from the cells after 4 or 16 h treatment with the electrophilic compounds using the RNeasy Mini Kit (Qiagen; Hilden, Germany) and converted into cDNA with RevertAid reverse transcriptase (Thermo Fisher Scientific; Waltham, MA, USA). For analysis of gene expression, qPCR was performed in a CFX Connect cycler using SsoAdvanced Universal SYBR Green Supermix (Bio-Rad Laboratories; Munich, Germany). PCR amplicons were quantified by the CFX Connect software, version 3.1, and results were computed as fold changes after normalization to the reference gene, encoding hypoxanthine-guanine phosphoribosyltransferase (HPRT1). Primers were synthesized by Thermo Fisher Scientific; their sequences are given in Table 1.

### 2.4. SDS-PAGE and Immunoblot Analysis

SDS-PAGE and immunoblotting were performed as previously described [26], with some modifications. Cells were either lysed in RIPA buffer supplemented with HALT protease inhibitor cocktail (Thermo Fisher Scientific), or cytoplasmic and nuclear fractions of the cells were obtained using the Nuclear Extract Kit (Active Motif; Carlsbad, CA, USA). Following determination of protein concentrations by the BCA protein assay (Thermo Fisher Scientific), proteins were run on SDS-polyacrylamide gels and electroblotted onto PVDF membranes (Carl Roth). Equal loading and blotting of the proteins were confirmed by staining with 0.1% Ponceau S solution in 5% acetic acid. Detection of Nrf2 protein bands occurred with primary (rabbit monoclonal anti-Nrf2; #12721; Cell Signaling Technology; Danvers, MA, USA) and secondary (goat anti-rabbit IgG-HRPO; #111-035-144; Dianova; Hamburg, Germany) antibodies, and SuperSignal West Pico and Femto (Thermo Fisher Scientific), using a ChemiDoc^TM^ MP analyzer and Image Lab^TM^ software, version 5.2.1 (Bio-Rad Laboratories). Relative Nrf2 levels were calculated upon normalization to total protein loading in each lane on Ponceau S-stained membranes, as it has been proposed for the reliable quantitation of Western blots [28]. 

To evaluate the quality of cell fractionation, the nuclear marker protein p53 and the cytoplasmic marker protein fatty acid synthase (FAS) were detected using a rabbit monoclonal anti-p53 antibody (#2527; Cell Signaling Technology) and a mouse monoclonal anti-FAS antibody (#sc-55580; Santa Cruz Biotechnology; Dallas, TX, USA), respectively. These marker proteins were chosen according to their primary intracellular localization in human cell lines, as depicted at The Human Protein Atlas website (https://www.proteinatlas.org/).

### 2.5. Statistical Analysis

Means were calculated from three independent experiments, and error bars represent standard error of the mean (S.E.M.). Statistical analysis was done using GraphPad PRISM software, version 8.0.1 (GraphPad Software; San Diego, CA, USA). As the number of n = 3 replicates is too small for establishing an underlying normal (Gaussian) distribution, the non-parametric Friedman test recommended for analysis of repeated matched measures was used for a general comparison of the ranks, and this was followed by post hoc testing of specific sample pairs by the Dunn test. Values of *p* < 0.05 were considered statistically significant.

## 3. Results and Discussion

### 3.1. Cytotoxicity of Selenite and the Applied Electrophilic Compounds in HepG2 Cells

**Figure 1:**
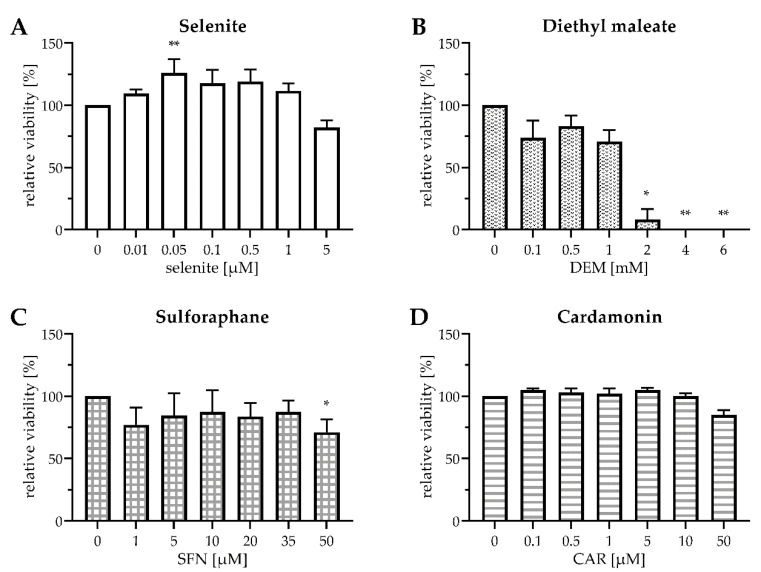
Viability of HepG2 cells after exposure to selenite and electrophiles for 24 h. Cells were cultured for 16 h in serum-free medium. Thereafter, they were cultured for another 24 h in serum-free medium containing sodium selenite at the indicated concentrations (**A**) or in serum-free medium containing 0.1 µM selenite and DEM (**B**), SFN (**C**), or CAR (**D**) at the indicated concentrations. Cell viability was assessed either by MTT assay (**A**–**C**) or by Neutral Red assay (**D**). Relative values were calculated by setting the viability of mock-treated cells to 100%. Three independent experiments were performed, each in triplicate; the data represent means ± S.E.M. Statistical analysis was done using the Friedman test and Dunn post hoc test, with * *p* < 0.05 vs. mock-treated cells and ** *p* < 0.01 vs. mock-treated cells.

First, we checked for potential cytotoxicity of the applied substances in HepG2 cells (Figure 1), as both selenite and electrophiles may exert oxidative stress at high doses.

Sodium selenite was chosen as the Se source for the experiments. A previous study comparing the metabolism of different Se compounds in HepG2 cells found that selenite at a Se-adequate dose of 0.1 µM most efficiently stimulated the activity of the antioxidant selenoenzyme GPx1 and the biosynthesis of selenoprotein P (SELENOP) [29]. Both selenoproteins are abundantly expressed in hepatocytes [30]; GPX1 and SELENOP transcripts together constitute ~70% of total selenoprotein mRNA in mouse liver [31]. Compared to Se deficiency, selenite at concentrations of up to 1 µM slightly increased the survival of HepG2 cells by trend (Figure 1A), probably through stimulation of their metabolic activity and/or proliferation, as it has been reported before for Caco-2 cells [13]. As selenite was not cytotoxic up to 1 µM, this concentration was chosen to mimic Se-supranutritional conditions, in addition to the Se-adequate concentration of 0.1 µM selenite.

DEM and SFN exerted a slight cytotoxic effect on HepG2 cells after 24 h of exposure, even at low doses. At 1 mM DEM and 50 µM SFN, the concentrations chosen for the subsequent experiments, ~75% of the HepG2 cells were viable after 24 h incubation (Figure 1B,C). Treatment with 1 mM DEM has been shown before to increase mRNA levels of the Nrf2 target gene heme oxygenase 1 (HO-1; gene name: HMOX1) in HepG2 cells [26]. Even SFN concentrations as low as 5–10 µM were demonstrated to be capable of inducing Nrf2 signalling in cultured human and rodent cells [13,24,32]. Doses of up to 30 µM SFN have been reported before to upregulate mRNA levels of the Nrf2 target gene glutathione-S-transferase A1 (gene name: GSTA1), with no measurable cytotoxicity [33]. 

As CAR appeared to interfere with the MTT assay, cell viability was assessed using the Neutral Red assay, revealing that CAR was not cytotoxic at doses up to 10 µM. After treatment with 50 µM CAR for 24 h, ~80% of the HepG2 cells were viable (Figure 1D), well in accordance with previous data from Caco-2 cells [22]. As 50 µM CAR triggered a robust induction of HMOX1 and other Nrf2 target genes in Caco-2 cells [22], this concentration was chosen for further experiments. 

### 3.2. Exposure of HepG2 Cells to Electrophiles Induces Rapid Nrf2 Stabilization and Nuclear Translocation that Is Largely Independent of the Cellular Se Status

Earlier studies on the interplay between Se and Nrf2 either did not explore cellular Nrf2 protein levels, thus relying on indirect effects on Nrf2 signalling [14,15,17], or did not observe the influence of Se on Nrf2 nuclear translocation despite synergistic effects of Se and Nrf2-activating isothiocyanates on Nrf2 target genes [13]. However, these early studies were somewhat hampered by a long-lasting dispute on the migratory behaviour of Nrf2 in reducing polyacrylamide gels and on the reliability of the antibodies used for Nrf2 detection in immunoblots [34]. In order to investigate Nrf2 protein levels and localization in HepG2 cells, we here made use of the D1Z9C rabbit monoclonal antibody (#12721; Cell Signaling Technology) that has been found to recognize Nrf2 in SFN-treated HepG2 cells with high specificity at an apparent molecular mass of ~100 kDa [32]. This is now thought to be the biologically relevant Nrf2 species, even though its apparent mass is considerably higher than the ~68 kDa predicted from human Nrf2 mRNA [32,34].

**Figure 2:**
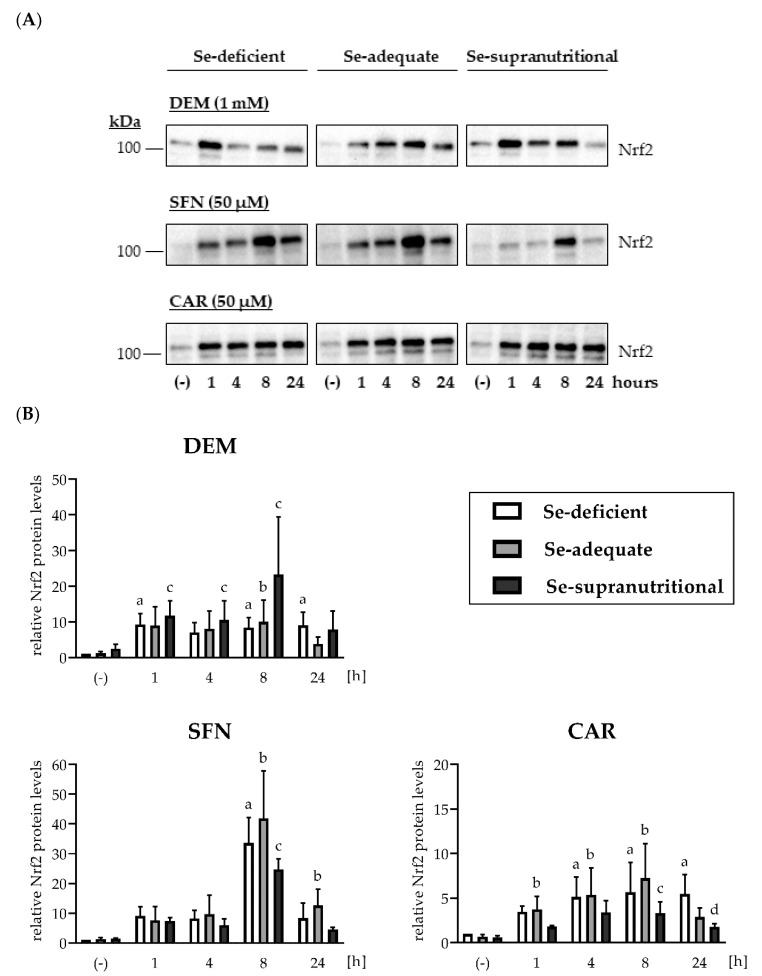
Induction of Nrf2 upon exposure of HepG2 cells to electrophiles. Cells were cultured for 16 h in serum-free medium without added Se (Se-deficient) or containing 0.1 µM selenite (Se-adequate) or 1 µM selenite (Se-supranutritional). Thereafter, the cells were cultured for the indicated time in serum-free medium with the three different Se levels and 1 mM DEM, 50 µM SFN, or 50 µM CAR, respectively. As solvent control, the cells were treated for 1 h with 0.1% DMSO (the lanes for the solvent control were marked with (-)). Nrf2 protein levels were detected by immunoblotting after cell lysis with RIPA buffer. (**A**) Each blot is representative of three independent experiments. (**B**) Relative Nrf2 protein levels as assessed by densitometric analysis of the immunoblots normalized against Ponceau S-stained protein bands; the data represent means ± S.E.M. Statistical analysis was done using the Friedman test and Dunn post hoc test, with statistical significance at *p* < 0.05: (a) significantly different from Se-deficient solvent control, (b) significantly different from Se-adequate solvent control, (c) significantly different from Se-supranutritional solvent control, (d) significantly different from Se-deficient CAR (24 h).

Nrf2 protein levels were low and barely detectable in HepG2 cells cultured under basal non-stressed conditions (Figure 2 and Figure S1A). The Se status alone did not affect cellular Nrf2 levels: Neither Se deficiency nor supranutritional Se supply resulted in elevated Nrf2 levels, as compared to HepG2 cells grown in Se-adequate medium (Figure 2 and Figure S1A). In contrast, each of the applied electrophiles induced a rapid increase in Nrf2 within 1 h of treatment that was also largely independent of the cellular Se status. Among the three electrophiles, only SFN elicited pronounced time course-dependent changes, with a peak in Nrf2 levels at 8 h of treatment. In contrast, the DEM- and the CAR-induced increases in Nrf2 levels remained more stable over the 24 h of exposure (Figure 2 and Figure S1A).

**Figure 3:**
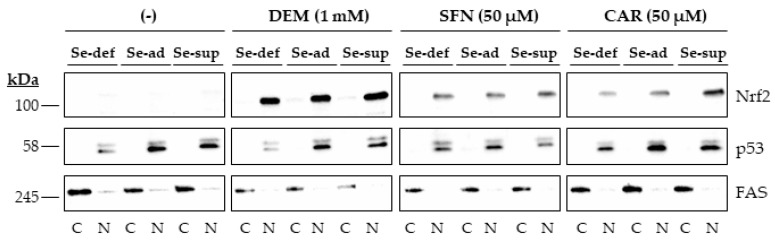
Nuclear accumulation of Nrf2 upon exposure of HepG2 cells to electrophiles. Cells were cultured for 16 h in serum-free medium without added Se (Se-deficient) or containing 0.1 µM selenite (Se-adequate) or 1 µM selenite (Se-supranutritional). Thereafter, the cells were cultured for 1 h in serum-free medium with the three different Se levels and 0.1% DMSO, 1 mM DEM, 50 µM SFN, or 50 µM CAR, respectively (the lanes for the solvent control were marked with (-)). Thereafter, cytoplasmic (C) and nuclear (N) fractions were prepared, and Nrf2 protein levels were detected by immunoblotting. The blots were then reprobed with antibodies against the nuclear marker protein p53 and the cytosolic marker protein FAS. Each blot is representative of three independent experiments.

Nrf2 accumulated in the nucleus of HepG2 cells upon 1 h of exposure to the electrophiles. Again, this occurred independently of the Se status, as no differences in response were observed in cells grown under Se-deficient, Se-adequate, and Se-supranutritional conditions. In contrast, very little Nrf2 was detected in the nucleus of cells treated with the solvent control DMSO (Figure 3 and Figure S1B). 

### 3.3. Electrophile-Mediated Induction of Nrf2 Target Genes Is Marginally Affected by the Se Status of HepG2 Cells

Next, we tested for the influence of Se status and of the electrophilic compounds on mRNA levels of prototypical Nrf2 target genes implicated in adaptive responses to cellular stress: HO-1 (gene name: HMOX1) catalyses the breakdown of heme into Fe^2+^, biliverdin, and carbon monoxide (CO); the latter two degradation products have been implicated in positive effects of HO-1, such as antioxidant, anti-apoptotic, and anti-inflammatory actions [35]. TrxR1 (gene name: TXNRD1) is crucial for cellular redox homeostasis, antioxidant defence, and redox signalling. By regenerating oxidized thioredoxin, TrxR1 provides oxidoreductases such as methionine sulfoxide reductases, ribonucleotide reductase, and some peroxiredoxin isoforms with reducing equivalents [36,37]. Glutathione-S-transferase A1 (gene name: GSTA1) belongs to a large family of glutathione-conjugating enzymes that participate in phase II metabolism of xenobiotics [38]. Sequestosome-1/p62 (gene name: SQSTM1) targets protein aggregates and individual proteins, including the Nrf2 repressor Keap1, for autophagy. The p62-mediated autophagic degradation of Keap1 results in prolonged Nrf2 activation/stabilization [6,7].

**Figure 4:**
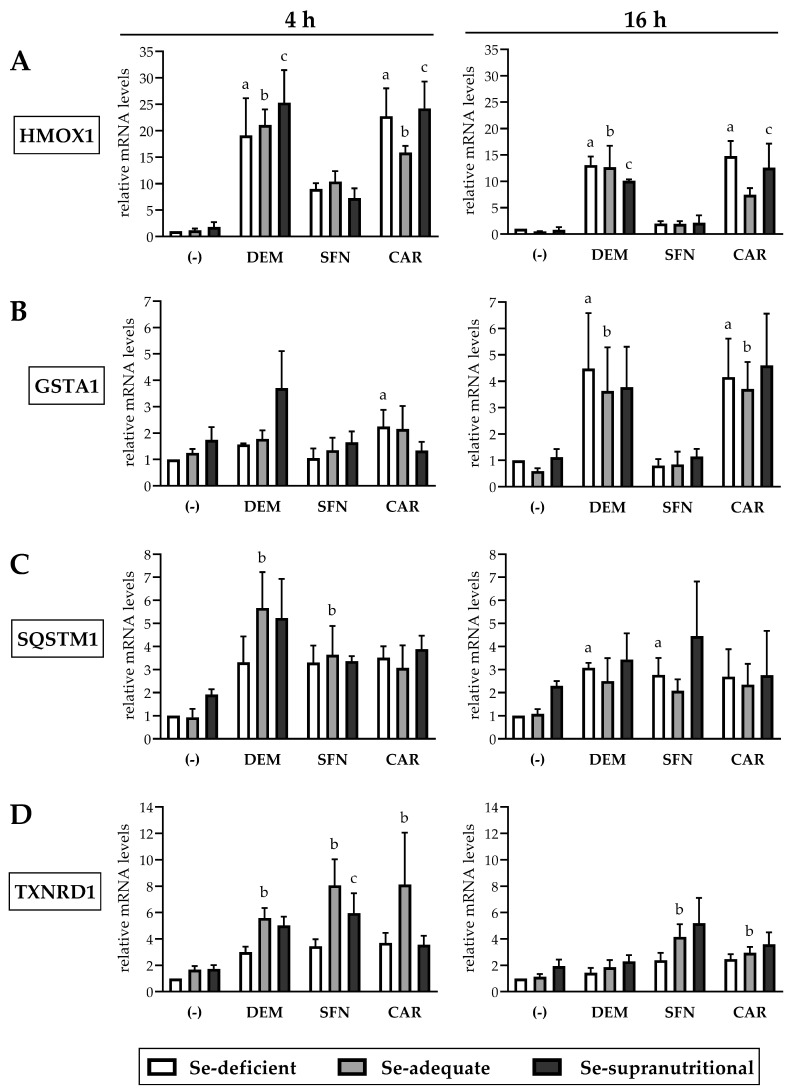
Electrophile-induced upregulation of Nrf2 target gene mRNAs is largely independent of the Se status of HepG2 cells. The cells were cultured for 16 h in serum-free medium without added Se (Se-deficient) or containing 0.1 µM selenite (Se-adequate) or 1 µM selenite (Se-supranutritional). Thereafter, cells were cultured for 4 h or 16 h in serum-free medium with the three different Se levels and 1 mM DEM, 50 µM SFN, or 50 µM CAR, respectively. As solvent control, the cells marked with (-) were treated with 0.1% DMSO. Relative mRNA levels of the Nrf2 targets HMOX1 (**A**), GSTA1 (**B**), SQSTM1 (**C**), and TXNRD1 (**D**) were determined by qRT-PCR, with normalization against HPRT1. Three independent experiments were performed; the data represent means ± S.E.M. Statistical analysis was done using the Friedman test and Dunn post hoc test, with statistical significance at *p* < 0.05: (a) significantly different from Se-deficient solvent control, (b) significantly different from Se-adequate solvent control, (c) significantly different from Se-supranutritional solvent control.

HMOX1 mRNA levels in HepG2 cells were neither altered at Se-deficient nor at Se-supranutritional conditions as compared to Se-adequate conditions (Figure 4A). This is in contrast to early studies that reported induction of HO-1 at both the mRNA and protein level in the liver of mice and rats subjected to a Se-deficient diet [15,16]. Selenite has also been reported to increase HO-1 levels in A549 human lung carcinoma cells via activation of Nrf2, even though this occurred at a high, cytotoxic dose of 6 µM and was accompanied by selenite-induced ROS generation [39]. On the other hand, our observations are well in accordance with data from a recent study, showing equal HO-1 levels in the liver of mice fed Se-deficient, Se-adequate, or Se-supranutritional diets [21]. 

Each of the three electrophiles induced a fast and strong increase in HMOX1 mRNA levels in HepG2 cells already after 4 h of exposure, which was somewhat attenuated after 16 h. At both time points, the effect of DEM and CAR on HMOX1 mRNA levels was more pronounced than that elicited by SFN. Neither DEM- nor SFN-mediated induction of HMOX1 was affected by the cellular Se status. The CAR-mediated induction of HMOX1 was, by trend, higher at Se-deficient and Se-supranutritional conditions, as compared to cells cultivated in Se-adequate medium (Figure 4A). This is in line with our previous observation that Se deficiency augmented the CAR-induced increase in HO-1 levels in Caco-2 cells [22].

GSTA1 mRNA levels increased upon DEM and CAR treatment of HepG2 cells, whereas SFN did not affect the gene expression of GSTA1. In contrast to HMOX1, induction of GSTA1 required a longer exposure to the electrophiles: GSTA1 mRNA levels were slightly elevated after 4 h but increased ~4-fold after 16 h of exposure to DEM or CAR (Figure 4B). This outcome was unexpected, as SFN has been shown before to induce a rapid ~3-fold increase in GSTA1 mRNA levels in HepG2 cells after only 30 min incubation that lasted for up to 18 h [33]. On the other hand, a delayed response of GSTA1 expression is not unusual; for example, treatment of HepG2 cells with the tyrosine kinase inhibitor lapatinib for 24 h has recently been reported to stimulate the transcription of GSTA1 und other glutathione-related enzymes via Nrf2 activation [40]. Neither the DEM- nor the CAR-mediated induction of GSTA1 after 16 h exposure was affected by the cellular Se status. After 4 h of exposure to DEM, GSTA1 mRNA levels were by trend higher in the Se-supranutritional cells, as compared to the Se-deficient and the Se-adequate groups (Figure 4B).

Treatment of HepG2 cells with DEM, SFN, or CAR resulted in similar increases in SQSTM1 mRNA levels, both after 4 h and after 16 h, and there was no statistically significant influence of the cellular Se status (Figure 4C). Thus, Se deficiency or selenite supplementation presumably did not affect the non-canonical pathway of Nrf2 activation/stabilization via p62/Keap1, in agreement with the observed time course of electrophile-induced increases in Nrf2 protein levels that was also largely independent of the cellular Se status (Figure 2).

TrxR1 is one of the two selenoenzymes that are also encoded by Nrf2 target genes [12,19]. Each of the three electrophiles caused a ~3-fold increase in TXNRD1 mRNA levels in Se-deficient HepG2 cells after 4 h treatment, whereas selenite alone did not affect TXNRD1 gene expression. There was a synergistic effect after combined exposure to selenite and the electrophiles with ~6–8-fold increases in TXNRD1 mRNA levels, particularly in the Se-adequate compared to the Se-deficient cells (Figure 4D). Similar results were found in a previous study that explored the interplay of Se and SFN in HepG2 cells. The authors concluded that Se is not capable of inducing TXNRD1 gene expression, but it may delay the degradation of SFN-induced TXNRD1 mRNA [14]. As in HepG2 cells, TXNRD1 mRNA levels in other human cell lines, such as Caco-2, HEK293, and LNCaP, were reported by us and others to not respond to changes in Se concentration of culture media, whereas TrxR1 protein levels and enzymatic activity were elevated in Se-supplemented cells [13,14,22,41]. 

Taken together, all three electrophilic compounds caused an enhanced expression of Nrf2 target genes, although with differences regarding extent and time course of their induction. Most likely, such differences might be explained by additional signalling pathways beyond Nrf2 that are affected by the individual electrophiles. For example, CAR has been reported to increase levels of the transcription factor peroxisome proliferator-activated receptor alpha (PPAR-α) in HepG2 cells and to interfere with the activity of the transcription factors sterol regulatory element-binding protein-1 (SREBP-1) and nuclear factor κB (NF-κB) [42,43]. 

Most important, we did not observe a statistically significant influence of the cellular Se status on the electrophile-induced upregulation of any of the four Nrf2 target genes explored here. 

### 3.4. Selenite-Induced Upregulation of Selenoprotein mRNAs Is Marginally Affected by Electrophiles

Limited bioavailability of Se under Se-deficient conditions dictates a cellular “selenoprotein hierarchy”, characterized by sustained biosynthesis of vital “housekeeping” selenoproteins at the expense of “stress-regulated” selenoproteins. This is predominantly maintained through switching off the incorporation of Sec into dispensable selenoproteins during ribosomal translation, but a few selenoprotein mRNAs are also downregulated in Se deficiency. In particular, GPx1 (gene name: GPX1) and selenoprotein W (gene name: SELENOW) rank very low in the “selenoprotein hierarchy” and are considered as good biomarkers for Se status, as their mRNA and protein levels decrease likewise in cells cultured in Se-poor medium and in animals fed a Se-deficient diet [30,41,44,45]. 

**Figure 5:**
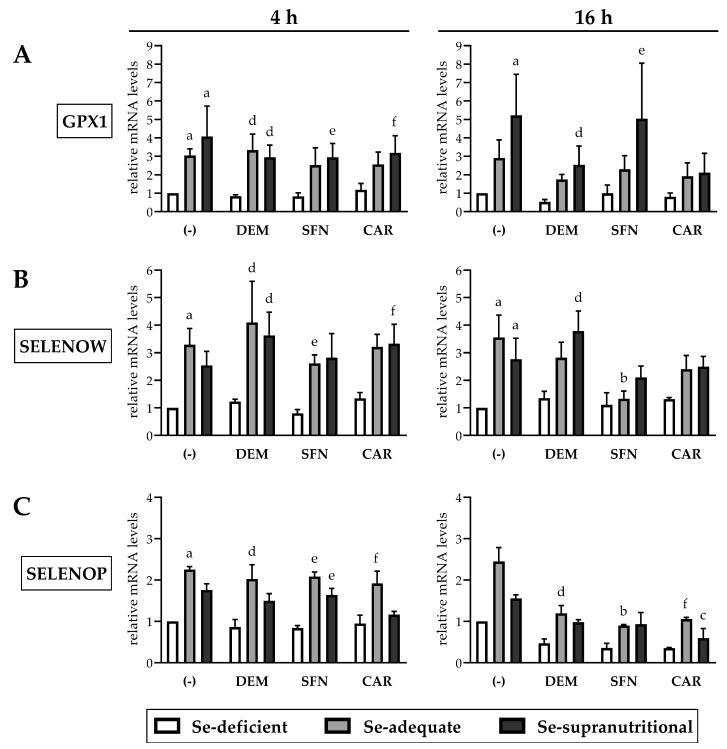
Impact of selenite and electrophiles on the mRNA levels of selected selenoproteins in HepG2 cells. Treatment of the cells and qRT-PCR analysis occurred as described for Figure 4 (Relative mRNA levels of GPX1 (**A**), SELENOW (**B**), SELENOP (**C**)). Statistical significance (*p* < 0.05): (a) significantly different from Se-deficient solvent control, (b) significantly different from Se-adequate solvent control, (c) significantly different from Se-supranutritional solvent control, (d) significantly different from Se-deficient DEM-treated cells, (e) significantly different from Se-deficient SFN-treated cells, (f) significantly different from Se-deficient CAR-treated cells.

As expected, GPX1 and SELENOW gene expression was higher in the Se-adequate and in the Se-supranutritional HepG2 cells, as compared to the Se-deficient group. There were no statistically significant differences in GPX1 and SELENOW mRNA levels between the Se-adequate and the Se-supranutritional group (Figure 5A,B). This points to saturation of the gene expression response for the selenoproteins already under Se-adequate conditions, similar to findings from animal experiments [45]. None of the applied electrophiles affected the selenite-mediated induction of the two selenoprotein mRNAs in HepG2 cells after short-term exposure for 4 h. However, GPX1 and SELENOW mRNA levels were, by trend, lower in the Se-adequate and the Se-supranutritional cells treated for 16 h with CAR than in the respective solvent control groups (Figure 5A,B). Similarly, we have observed before that CAR attenuated the selenite-induced increase in GPX1 mRNA and protein levels in Caco-2 cells [22]. Besides CAR, DEM lowered the selenite-induced increase in GPX1 gene expression, and SFN lowered the selenite-induced increase in SELENOW gene expression in HepG2 cells after 16 h treatment (Figure 5A,B).

In addition to GPX1 and SELENOW, mRNA levels of selenoprotein P (gene name: SELENOP), one of the most abundantly expressed selenoproteins in the liver [31], were explored. The plasma Se transporter SELENOP is primarily secreted from hepatocytes. SELENOP ranks above GPx1 in the selenoprotein hierarchy, as liver Se is redistributed from GPx1 to SELENOP in Se deficiency to maintain Se supply of the brain and some endocrine tissues [10,30]. Se-deficient HepG2 cells show suppressed SELENOP secretion [27,29,46], while data on SELENOP gene expression are conflicting: Supplementation of Se-deficient HepG2 cells with selenite has been reported to result in elevated, unaltered, and even decreased SELENOP mRNA levels [27,29,41,46]. Thus, SELENOP mRNA levels cannot be considered as a reliable biomarker of Se status, in contrast to the GPX1 and SELENOW mRNA levels. Here, we observed an ~2-fold higher SELENOP gene expression in Se-supplemented HepG2 cells, as compared to the Se-deficient group. Short-term exposure of HepG2 cells to the electrophiles did not affect SELENOP mRNA levels, whereas the selenite-induced increase in SELENOP gene expression was suppressed after 16 h treatment with DEM, SFN, or CAR (Figure 5C). 

As neither the SELENOP nor the GPX1 and SELENOW promoter regions are known to contain ARE/EpRE sequences for binding of Nrf2, our data may point to a post-transcriptional, selenoprotein mRNA-destabilizing effect of the electrophiles. 

## 4. Conclusions

Both dietary phytochemicals activating Nrf2 signalling and the micronutrient selenium are thought to be beneficial for human health, in part through supporting adaptive responses to oxidative stress as indirect “antioxidants” [1,47]. The interplay between the cellular Se status and Nrf2 signalling appears to be primarily mediated via the selenoenzyme TrxR1 that itself is an Nrf2 target gene [19]. A compensatory induction of Nrf2 target genes in Se deficiency has been reported before [15,16,17,18]; however, this was questioned in some recent studies [20,21,22]. Here, we found no evidence that alterations in Se status resulted in an increase in cellular Nrf2 levels, Nrf2 nuclear translocation or induction of Nrf2 target genes in HepG2 cells cultured either in Se-deficient, Se-adequate, or Se-supranutritional medium. Moreover, the induction of Nrf2 signalling following exposure of HepG2 cells to three Nrf2-activating electrophilic compounds (DEM, SFN, and CAR) was largely independent of the cellular Se status (Figure 2, Figure 3 and Figure 4). In order to explain such controversial and unexpected findings, it has recently been hypothesized that short-term Se deficiency may not be efficient enough to provoke an Nrf2 response [44]. Consequently, the interplay between Se and Nrf2 might then be particularly important under experimental conditions of prolonged strict Se deficiency or genetic disruption of selenoprotein biosynthesis, but physiologically less relevant for humans, as most people are nowadays at very low risk of developing a severe Se deficiency [47]. The dietary Se supply may, thus, not affect Nrf2 signalling in healthy and well-nourished humans. This is further illustrated by the results of a Danish intervention trial that found no alterations in Nrf2 target gene expression in isolated leucocytes of the study participants after supplementation of their usual diet with Se at supranutritional doses (300 and 480 µg Se/day) [48].

## Figures and Tables

**Table 1:** Primers (5′-3′) used for qRT-PCR analysis.

Gene	Gene-ID	Forward Primer	Reverse Primer
GPX1	NM_000581	caaccagtttgggcatcag	tctcgaagagcatgaagttgg
GSTA1	NM_145740	ctacgtcgaggagcttgactc	cttcttcactgtgggcaggt
HMOX1	NM_002133	agactgcgttcctgctcaac	ggctctggtccttggtgtc
HPRT1	NM_000194	ggggacataaaagtaattggtggag	ctgaccaaggaaagcaaagtctg
SELENOP	NM_005410	aactgctctctcacgactctc	agcatttggtgctcctggtt
SELENOW	NM_003009	cgtggacacagaaagcaagtt	gagaggggctgggtcaag
SQSTM1	NM_003900	agctgccttgtacccacatc	cagagaagcccatggacag
TXNRD1	NM_182729	ttggaatccaccctgtctgt	catccacactggggcttaac

## Data Availability

The data presented in this study are available within the article and its Supplementary Material.

## Supplementary Materials

The following are available online at https://www.mdpi.com/2076-3921/10/2/167/s1, Figure S1A: Time-dependent changes in Nrf2 protein levels after exposure of HepG2 cells to electrophiles, Figure S1B: Nuclear accumulation of Nrf2 after exposure of HepG2 cells to electrophiles.
