# Supplementary material for: Activation of Nrf2 by Electrophiles Is Largely Independent of the Selenium Status of HepG2 Cells
Source: Antioxidants (Basel). 2021 Jan 23;10(2):167. doi: 10.3390/antiox10020167 (PMC7911449; doi:10.3390/antiox10020167)
Supplement: Supplementary file 1 [file antioxidants-10-00167-s001.pdf]

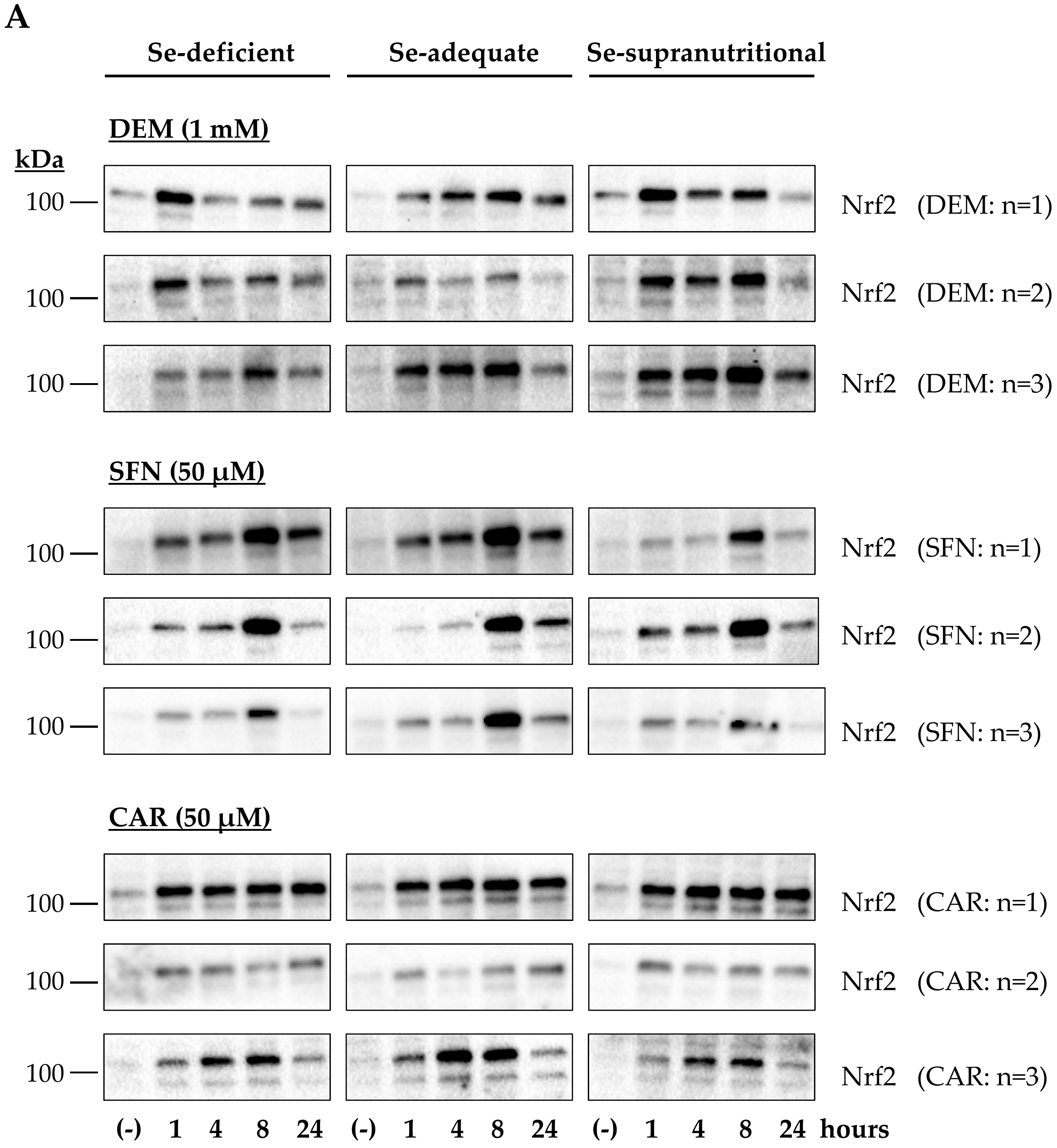

Figure S1A. Time-dependent changes in Nrf2 protein levels after exposure of HepG2 cells to electrophiles. HepG2 cells were treated for the indicated times with DEM, SFN or CAR in serum-free medium containing no Se (Se-deficient), 0.1 µM selenite (Se-adequate) or 1 µM selenite (Se-supranutional). DMSO served as solvent control (-). For the experimental details, see Figure 2. The immunoblots of n=3 independent experiments are shown.

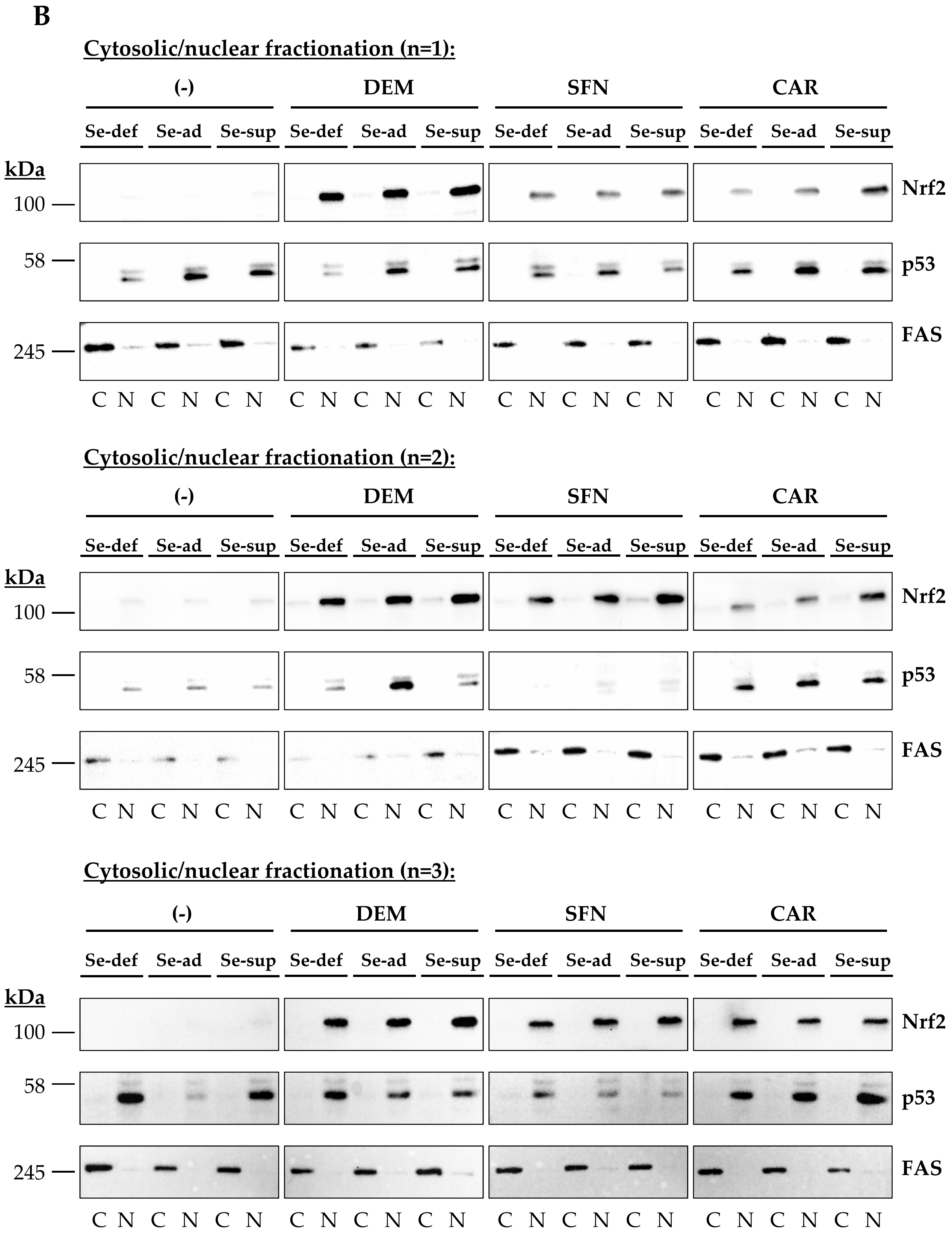

Figure S1B. Nuclear accumulation of Nrf2 after exposure of HepG2 cells to electrophiles.
HepG2 cells were treated for 1 h with DEM, SFN or CAR in serum-free medium containing no Se (Se-deficient), 0.1 µM selenite (Se-adequate) or 1 µM selenite (Se-supranutional). DMSO served as solvent control (-). For the experimental details, see Figure 2. The immunoblots of n=3 independent experiments are shown.
